# Resilience and Grief-Related Functional Impairment in Paraguayan Adults With Recent Significant Loss: A Cross-Sectional Study

**DOI:** 10.31083/AP51829

**Published:** 2026-06-28

**Authors:** Julio Torales, Juliana Bae, Melissa Medina, Fiorella Velázquez, Maia Leguizamón, Eva Giménez-Legal, Marcelo O´Higgins, Tomás Caycho-Rodríguez, João Mauricio Castaldelli-Maia, Antonio Ventriglio, Iván Barrios

**Affiliations:** ^1^Research Group on Epidemiology of Mental Disorders, Psychopathology and Neurosciences, School of Medical Sciences, Universidad Nacional de Asunción, 216001 San Lorenzo, Paraguay; ^2^Vice-Rectorate for Research and Postgraduate Studies, Universidad de Los Lagos, 5290000 Osorno, Chile; ^3^Research Department, School of Health Sciences, Universidad Sudamericana, 120101 Salto del Guairá, Paraguay; ^4^Department of Psychology, Universidad Científica del Sur, 15067 Lima, Perú; ^5^Department of Psychiatry, University of São Paulo, São Paulo, SP 05403-010, Brazil; ^6^Department of Clinical and Experimental Medicine, University of Foggia, 71122 Foggia, Italy; ^7^Department of Biostatistics, School of Medical Sciences, Santa Rosa Campus, Universidad Nacional de Asunción, 021804 Santa Rosa del Aguaray, Paraguay

**Keywords:** bereavement, resilience, grief, functional impairment, Paraguay

## Abstract

**Background:**

Bereavement is a common life experience that can lead to diverse psychological outcomes, ranging from adaptive coping to significant functional impairment. While resilience has been identified as a key factor in psychological adaptation after loss, the relationship between resilience and grief-related functional impairment remains insufficiently explored in many cultural contexts, particularly in Latin America. This study examined the association between resilience and grief-related functional impairment in Paraguayan adults who had experienced a recent significant loss.

**Methods:**

A cross-sectional study was conducted using a non-probabilistic convenience sample of 215 Paraguayan adults aged 18–40 years. Data were collected through an online questionnaire including sociodemographic variables and the Connor–Davidson Resilience Scale (CD-RISC-25). Participants who reported the loss of a significant person during the previous two years additionally completed the Grief Impairment Scale and contextual questions related to the loss. Descriptive, bivariate, and multivariable analyses were performed using RStudio.

**Results:**

Overall, 59.5% of participants reported having experienced a significant loss within the previous two years. Mean resilience scores did not differ significantly between individuals with and without recent loss (*p *= 0.773). Within the bereaved subsample (n = 128), resilience was significantly associated with educational level (*p *= 0.020) and religiosity (*p *= 0.027). Grief-related functional impairment was associated with sex (*p *= 0.048), type of relationship with the deceased (*p *= 0.035), time since loss (*p *= 0.023), perceived social support (*p *= 0.049), and psychotropic medication use (*p *= 0.016). Multivariable models confirmed educational level and religiosity as predictors of resilience.

**Conclusions:**

Resilience scores did not differ significantly between individuals with and without recent bereavement at the time of assessment, even among individuals experiencing functional difficulties related to grief. Contextual factors such as religiosity and social support appear to play an important role in shaping bereavement outcomes. These findings highlight the importance of assessing both adaptive psychological resources and functional impairment in the evaluation of grief.

## Main Points

1. Resilience scores did not differ significantly between individuals who experienced a recent significant loss and those who had not, at the time of assessment.

2. In the bereaved subsample, resilience was significantly associated with educational level and religiosity, highlighting the potential role of psychosocial and cultural factors in adaptive responses to loss.

3. Grief-related functional impairment was associated with several contextual factors, including sex, relationship to the deceased, time since loss, perceived social support, and psychotropic medication use.

4. The findings support the view that resilience and grief-related functional impairment represent distinct psychological processes that may coexist following bereavement.

5. Assessing both resilience and functional impairment may provide a more comprehensive understanding of bereavement outcomes and help identify individuals who could benefit from targeted psychosocial support.

## 1. Introduction

Bereavement is a complex emotional, cognitive, and behavioral response to the loss of a significant person. Although grief is often considered a natural and adaptive process that allows individuals to gradually reorganize their lives and restore psychological balance, in some cases it may lead to substantial functional difficulties affecting cognition, physical health, daily activities, occupational responsibilities, and interpersonal relationships [1,2,3,4]. The clinical relevance of these impairments has prompted the development of instruments designed to assess grief-related functional consequences, such as the Grief Impairment Scale (GIS), which evaluates the degree to which grief interferes with everyday functioning [5]. Recent studies have supported the psychometric properties and cross-cultural applicability of the Spanish version of the GIS in Latin American populations [6,7].

Among the psychological factors that may influence adaptation to loss, resilience has received considerable attention. Resilience is commonly defined as the capacity to adapt positively to adversity and to recover from stressful or traumatic experiences [8,9]. This multidimensional construct can be measured using instruments such as the Connor–Davidson Resilience Scale (CD-RISC-25), one of the most widely used tools for assessing resilience in clinical and community populations [9]. However, it is important to note that resilience is a context-dependent construct, and its operationalization may vary according to the nature of the adversity, the criteria used to define positive adaptation, and the characteristics of the population under study. In this regard, the CD-RISC-25 represents one specific and widely accepted approach to quantifying resilience within a particular conceptual and measurement framework. The Spanish version of the CD-RISC-25 has demonstrated adequate psychometric properties in several Spanish-speaking populations [10].

Previous research suggests that individuals with higher resilience may experience lower psychological distress after adverse life events and may be associated with more effective adaptation to stressful circumstances [8,11]. In the context of bereavement, resilience has been proposed as a potential protective factor that could mitigate emotional suffering and functional disruption. However, empirical findings remain heterogeneous, and most studies examining resilience and grief outcomes have been conducted in clinical samples or in high-income countries. Consequently, the relationship between resilience and grief-related functional impairment requires further exploration in diverse sociocultural contexts and in community-based populations.

In addition to individual psychological resources, several contextual and demographic factors may influence how individuals respond to the loss of a loved one. Characteristics such as the relationship to the deceased, the time elapsed since the loss, perceived social support, religiosity, and the use of psychotropic medication may shape the intensity of grief reactions and their impact on everyday functioning [12,13]. Sociodemographic characteristics including age, sex, marital status, and educational level may also contribute to variability in coping responses following bereavement.

In this context, the present study aimed to examine resilience and grief-related functional impairment among adults in Paraguay who had experienced a recent significant loss. Specifically, the study sought to compare resilience, as measured by CD-RISC-25 scores, between individuals with and without recent bereavement and to explore the association between resilience and grief-related functional impairment among those who had experienced a loss. Additionally, we examined sociodemographic and contextual factors associated with resilience and functional impairment related to grief.

## 2. Materials and Methods

### 2.1 Study Design

This study employed an observational, cross-sectional design with a quantitative approach. The aim was to examine resilience levels among adults living in Paraguay according to the experience of a recent significant loss and to explore the association between resilience and grief-related functional impairment among individuals who had experienced bereavement. A cross-sectional design allowed for the assessment of psychological adaptation and functional outcomes at a specific point in time and facilitated the examination of associations between resilience and grief-related functional impairment [14].

### 2.2 Population and Sampling

The target population consisted of adults residing in Paraguay. Participants were recruited through a non-probabilistic convenience sampling strategy using an open invitation disseminated through social media platforms, community groups, and institutional communication channels.

Data were collected through a self-administered online questionnaire hosted on Google Forms (Google LLC, Mountain View, CA, USA; https://docs.google.com/forms/), a freely accessible digital platform. The survey link was distributed through institutional mailing lists, social media, and messaging applications such as WhatsApp (WhatsApp LLC / Meta Platforms, Inc., Menlo Park, CA, USA; https://www.whatsapp.com/) and Telegram (Telegram Messenger Inc., Dubai, United Arab Emirates; https://telegram.org/). Prior to accessing the questionnaire, participants were informed about the objectives of the study, the voluntary nature of participation, and the confidentiality and anonymity of the collected data. Online surveys have been shown to be a reliable and efficient method for collecting behavioral and psychological data, producing results comparable to those obtained through traditional data collection methods [15].

All participants completed a questionnaire including sociodemographic information (age, sex, marital status, and educational level) and the Spanish version of the CD-RISC-25 [9,10]. Participants were then asked whether they had experienced the loss of a significant person within the previous two years. Those who reported no recent loss proceeded directly to the resilience scale, which assesses general perceived capacity to cope with adversity rather than responses to a specific recent stressor. Participants who reported a significant loss completed an additional module assessing contextual characteristics of the loss, including the relationship to the deceased, time elapsed since the most recent loss, perceived social support, self-perceived religiosity, regular religious or spiritual practice, and current use of psychotropic medication. These participants also completed the GIS, which measures the functional impact of grief [6,7].

The inclusion criteria were: being 18 years of age or older, currently residing in Paraguay, and providing informed consent to participate in the study. Questionnaires with more than 20% missing responses were excluded from the analysis to ensure data quality and internal consistency.

### 2.3 Variables

Several sociodemographic, bereavement-related, contextual, and psychological variables were collected to characterize the study population and to explore the relationship between resilience and grief-related functional impairment.

#### 2.3.1 Sociodemographic Variables

Sociodemographic information included age (continuous variable measured in years), sex (male, female, other), marital status (single, in a relationship, married, separated/divorced, or widowed), and educational level (primary education, secondary education, technical training, university education, or postgraduate education).

#### 2.3.2 Bereavement Status

Participants were asked whether they had experienced the loss of a significant person within the previous two years (yes/no), a timeframe selected to capture relatively recent bereavement experiences while allowing for variability in grief responses and functional adaptation. This variable determined the subsequent flow of the questionnaire. Participants who reported no recent loss proceeded directly to the resilience assessment, whereas those who reported a significant loss completed additional questions regarding the circumstances of the loss and the functional impact of grief.

#### 2.3.3 Psychological Measures

Resilience was assessed using the Spanish version of the CD-RISC-25 [9,10], and grief-related functional impairment was assessed using the GIS [5,6,7], as described in detail in section 2.4.

#### 2.3.4 Contextual Variables Related to Bereavement

Among participants who reported a recent significant loss, additional contextual variables were collected. These included the relationship to the deceased (parent, partner, child, sibling, grandparent, or other), time elapsed since the most recent loss (less than 6 months, 6–12 months, or 1–2 years), perceived social support (self-reported on a five-point scale ranging from 1 [none] to 5 [very high]), self-perceived religiosity (four-point scale ranging from not religious to very religious), regular religious or spiritual practice (yes/no), and current use of psychotropic medication (yes/no), including anxiolytics, antidepressants, or other prescribed psychotropic drugs. Perceived social support and religiosity were assessed using single-item self-report measures developed for this study and were not based on formally validated instruments.

These variables were included in the analyses to explore their potential association with resilience and grief-related functional impairment.

### 2.4 Measures

Data were collected using self-administered measures with established psychometric properties and widely used in psychological and psychiatric research.

#### 2.4.1 Connor–Davidson Resilience Scale (CD-RISC-25)

Resilience was assessed using the Spanish version of the CD-RISC-25, originally developed by Connor and Davidson [9] and later validated in Spanish-speaking populations [10]. This instrument was selected due to its widespread use in both clinical and community populations, its strong psychometric performance in Spanish-speaking samples, and its suitability for assessing general adaptive capacity across diverse contexts. The CD-RISC-25 consists of 25 items that measure the ability to adapt to adversity during the previous four weeks. Each item is rated on a five-point Likert scale ranging from 0 (not true at all) to 4 (true nearly all the time). Total scores range from 0 to 100, with higher scores indicating greater resilience [9,10].

#### 2.4.2 Grief Impairment Scale (GIS)

Grief-related functional impairment was assessed using the Spanish version of the GIS [6]. The GIS is a brief five-item instrument that evaluates the extent to which grief interferes with cognitive functioning, physical well-being, daily activities, and interpersonal relationships during the previous 30 days. Items are rated on a five-point scale ranging from 0 (never) to 4 (always), yielding total scores between 0 and 20. Higher scores indicate greater functional impairment associated with grief [5,6,7].

#### 2.4.3 Sociodemographic and Contextual Questionnaire

A brief questionnaire developed for this study was used to collect sociodemographic information and contextual variables related to bereavement, including marital status, educational level, perceived social support, religiosity, religious practice, and use of psychotropic medication.

### 2.5 Statistical Analysis

All statistical analyses were conducted using RStudio, version 2025.05.0+496 (Posit Software, PBC, Boston, MA, USA; https://posit.co) with the R statistical computing environment. Descriptive, bivariate, and multivariable analyses were performed in accordance with the study objectives and the characteristics of the variables.

Descriptive statistics were used to summarize the study variables. Continuous variables (age, CD-RISC-25 scores, and GIS scores) were described using means, standard deviations, and ranges. Categorical variables (sex, marital status, educational level, experience of significant loss, type of loss, time since loss, perceived social support, religiosity, religious practice, and use of psychotropic medication) were summarized using frequencies and percentages.

Bivariate analyses were conducted to examine differences between groups. Resilience scores (CD-RISC-25) were compared between participants with and without recent significant loss using Student’s *t* test or the Mann-Whitney U test, depending on the distribution of the variable, with normality assessed using the Kolmogorov-Smirnov test. Within the subsample of participants who reported a significant loss, additional comparisons were performed to evaluate differences in resilience and grief-related functional impairment across categorical variables such as type of relationship with the deceased, religiosity, and psychotropic medication use. For comparisons involving more than two groups, the Kruskal-Wallis test was applied.

Spearman’s correlation coefficient was used to explore the association between resilience scores and grief-related functional impairment. The normality of continuous variables was assessed using the Kolmogorov-Smirnov test.

Finally, multiple linear regression models were constructed to identify factors associated with resilience scores. In the full sample, variables showing significant associations in bivariate analyses were included as predictors. This selection approach was used to identify variables with preliminary evidence of association; however, it may have excluded potentially relevant factors that were not measured or did not reach statistical significance in bivariate analyses. In the subsample of participants who had experienced a significant loss, an additional model was estimated including grief-related functional impairment as a predictor. Standardized beta coefficients, standard errors, and 95% confidence intervals were reported. Statistical significance was set at *p *< 0.05. For categorical predictors included in the regression models, reference categories were selected based on conceptual relevance and the distribution of the data, typically using the highest or most common category as the reference group to facilitate interpretation of the regression coefficients, and the same coding approach was applied consistently across models.

No formal adjustment for multiple comparisons was applied, given the exploratory nature of the study and the aim of identifying potential associations for further investigation.

### 2.6 Sample Size

The minimum sample size was estimated based on previous studies reporting prevalence rates of significant bereavement or related symptoms in the general adult population ranging between 30% and 50% [2,16]. Assuming an expected proportion of 40% of individuals experiencing recent significant loss, a confidence level of 95%, and an absolute margin of error of 8%, the estimated minimum sample size was 144 participants.

This value was increased to allow subgroup comparisons and to account for potential exclusions due to incomplete responses. Data collection remained open until a final sample size was reached that allowed the planned bivariate and multivariable analyses, particularly within the subgroup of participants who had experienced bereavement [17,18].

## 3. Results

A total of 215 participants were included, with ages ranging from 18 to 40 years (mean = 23.3 ± 4.76 years). Women represented 73.0% of the sample, 75.8% were single, and 79.5% had a university-level education. Overall, 59.5% of participants reported having experienced a significant loss during the previous two years (Table 1).

**Table 1.** **Sociodemographic characteristics of the total sample (N = 215)**.

Variable		n	%
Sex			
	Female	157	73.0
	Male	58	27.0
Marital status			
	Single	163	75.8
	Not single	52	24.2
Educational level			
	Postgraduate	13	6.0
	University	171	79.5
	Technical	11	5.1
	Completed secondary education	20	9.3
Significant loss (last two years)			
	Yes	128	59.5
	No	87	40.5

Note. Sample size: N = 215. Percentages may not total 100 due to rounding.

Internal consistency of the scales was high. The CD-RISC-25 showed a Cronbach’s alpha of 0.958, while the GIS showed a Cronbach’s alpha of 0.877. In the total sample, the mean resilience score was 59.9 ± 23 points (observed range: 0–100, theoretical range: 0–100). Among participants who reported a significant loss, the mean GIS score was 5.21 ± 4.23 points (observed range: 0–16, theoretical range: 0–20). Descriptive statistics for individual items of the CD-RISC-25 and the GIS are presented in Tables 2,3.

**Table 2.** **Descriptive statistics for individual items of the CD-RISC-25 in the total sample (N = 215)**.

Item	M	SD	Min	Max	Skewness	Kurtosis
1	2.51	1.14	0	4	–0.64	2.77
2	2.58	1.43	0	4	–0.53	1.86
3	2.27	1.38	0	4	–0.26	1.84
4	2.35	1.22	0	4	–0.23	2.14
5	2.42	1.33	0	4	–0.43	2.06
6	2.34	1.36	0	4	–0.22	1.75
7	2.62	1.27	0	4	–0.69	2.47
8	2.45	1.28	0	4	–0.38	2.15
9	2.68	1.45	0	4	–0.74	2.14
10	2.56	1.34	0	4	–0.62	2.23
11	2.75	1.35	0	4	–0.83	2.44
12	2.56	1.35	0	4	–0.58	2.13
13	2.28	1.31	0	4	–0.31	1.97
14	2.07	1.17	0	4	–0.11	2.10
15	2.65	1.41	0	4	–0.78	2.28
16	1.90	1.30	0	4	0.18	1.95
17	2.41	1.29	0	4	–0.34	1.93
18	2.32	1.20	0	4	–0.15	1.98
19	2.22	1.27	0	4	–0.21	2.04
20	2.35	1.18	0	4	–0.38	2.29
21	2.29	1.36	0	4	–0.21	1.87
22	2.09	1.31	0	4	–0.12	1.92
23	2.22	1.30	0	4	–0.24	2.04
24	2.54	1.28	0	4	–0.59	2.35
25	2.48	1.34	0	4	–0.52	2.14

Note. Sample size: N = 215. Items are rated on a 5-point Likert scale ranging from 0 (not true at all) to 4 (true nearly all the time). CD-RISC-25, Connor–Davidson Resilience Scale; M, mean; SD, standard deviation; Min, minimum; Max, maximum. Skewness and kurtosis values are presented to describe distributional properties. Observed values are reported.

**Table 3.** **Descriptive statistics for individual items of the GIS in the bereaved subsample (n = 128)**.

Item	M	SD	Min	Max	Skewness	Kurtosis
1	1.21	0.97	0	3	0.29	2.05
2	1.22	1.11	0	4	0.45	2.07
3	0.80	1.09	0	4	0.97	2.71
4	0.91	0.89	0	3	0.70	2.70
5	1.06	1.09	0	4	0.74	2.75

Note. Sample size: n = 128. Items are rated on a 5-point scale ranging from 0 (never) to 4 (always). GIS, Grief Impairment Scale. Skewness and kurtosis values are presented to describe distributional properties. Observed values are reported.

Among participants who had experienced significant loss (n = 128), the most frequently reported relationship with the deceased was grandparent (45.3%), followed by other relationships (26.6%) and parent (19.5%). Most participants reported that the most recent loss had occurred between one and two years prior to the assessment (57.8%), whereas 24.2% had experienced the loss within the previous six months. The assessment focused on the most recent significant loss, and information on the number of losses experienced by each participant was not collected.

Regarding contextual variables, moderate perceived social support was the most frequently reported category (46.1%). In terms of religiosity, most participants described themselves as somewhat religious (58.6%). The majority reported not attending religious or spiritual ceremonies regularly (67.2%) and not using psychotropic medication (95.3%).

In the total sample, bivariate analyses showed no significant differences in resilience between participants with and without significant loss (*t* = 0.289; *p *= 0.773). Participants without loss had a mean resilience score of 60.5 [standard deviation (SD) = 22.9], whereas those with loss had a mean score of 59.5 (SD = 23.2). No statistically significant differences were found according to sex (*t* = −1.96; *p *= 0.051) or marital status (*t* = −1.41; *p *= 0.160), although descriptively higher resilience scores were observed among men than women.

Resilience differed significantly according to educational level (Kruskal-Wallis = 10.3; *p *= 0.016), with the highest scores observed among participants with completed secondary education.

Within the subsample of participants who had experienced significant loss, resilience differed significantly according to educational level (Kruskal-Wallis = 9.80; *p *= 0.020) and religiosity (Kruskal-Wallis = 9.18; *p *= 0.027). Higher resilience scores were observed among participants with completed secondary education and among those who described themselves as fairly religious. No significant associations were observed between resilience and sex (*p *= 0.107), marital status (*p *= 0.295), type of relationship with the deceased (*p *= 0.658), time since loss (*p *= 0.177), perceived social support (*p *= 0.070), religious attendance (*p *= 0.433), or psychotropic medication use (*p *= 0.395).

Grief-related functional impairment showed several significant associations. GIS scores were significantly higher among women than men (*t* = 1.99; *p *= 0.048). Significant differences were also found according to type of relationship with the deceased (Kruskal-Wallis = 10.3; *p *= 0.035), time since loss (Kruskal-Wallis = 7.53; *p *= 0.023), perceived social support (Kruskal-Wallis = 9.52; *p *= 0.049), and psychotropic medication use (*U* = 153; *p *= 0.016). Given that some *p*-values were close to the significance threshold and that no adjustment for multiple comparisons was applied, these findings should be interpreted with caution. Higher GIS scores were observed among participants who had lost a sibling or parent, among those reporting lower levels of perceived social support, and among those currently using psychotropic medication. No significant associations were found with marital status (*p *= 0.149), educational level (*p *= 0.127), religiosity (*p *= 0.384), or religious attendance (*p *= 0.404).

Correlation analyses showed no significant association between resilience and grief-related functional impairment (*rho* = −0.0996; *p *= 0.263). Likewise, age was not significantly correlated with resilience (*rho* = −0.0091; *p *= 0.919) or grief-related impairment (*rho* = 0.0995; *p *= 0.264).

In the multivariable model for the total sample, only educational level was included as a predictor of resilience. The model was statistically significant overall (*F* = 3.795; *p *= 0.011), although it explained a small proportion of the variance (adjusted *R^2^* = 0.038). Compared with participants with postgraduate education, those with completed secondary education showed significantly higher resilience scores (*β* = 22.97; 95% CI: 7.12–38.8; *p *= 0.005) (Table 4).

**Table 4.** **Multiple linear regression model for resilience (CD-RISC-25) in the total sample (N = 215)**.

Variable	*β*	95% CI	*p*
Intercept	52.39	40.00–64.70	<0.001
Secondary education	22.97	7.12–38.80	0.005
Technical education	8.52	–9.70–26.70	0.357
University education	6.24	–6.56–19.00	0.337

Note. Sample size: N = 215. Reference category: postgraduate education. Adjusted *R^2^* = 0.038. Durbin-Watson statistic = 2.29.

In the subsample of participants with significant loss, the multivariable model included educational level and religiosity. The model was statistically significant (*F* = 3.345; *p *= 0.004) and explained approximately 10% of the variance in resilience scores (adjusted *R^2^* = 0.100). Compared with postgraduate education, having a university degree was associated with lower resilience scores (*β* = −21.65; 95% CI: −42.2 to −1.10; *p *= 0.039). In terms of religiosity, being fairly religious was associated with higher resilience scores compared with being somewhat religious (*β* = 11.41; 95% CI: 1.96–20.9; *p *= 0.018) (Table 5).

**Table 5.** **Multiple linear regression model for resilience (CD-RISC-25) among participants with significant loss (n = 128)**.

Variable	*β*	95% CI	*p*
Intercept	77.26	56.9–97.6	<0.001
Secondary education	–3.06	–27.5–21.4	0.805
Technical education	–18.40	–43.4–6.62	0.148
University education	–21.65	–42.2 to –1.10	0.039
Fairly religious	11.41	1.96–20.9	0.018
Very religious	–5.66	–20.3–8.93	0.444
Not religious	–10.18	–24.5–4.11	0.161

Note. Sample size: n = 128. Reference categories: postgraduate education and somewhat religious. Adjusted *R^2^* = 0.100. No relevant multicollinearity was detected [Generalized Variance Inflation Factor (GVIF) ≈ 1.02]. Durbin-Watson statistic = 2.29.

## 4. Discussion

The present study examined the relationship between resilience and grief-related functional impairment in a sample of Paraguayan adults, comparing individuals who had experienced a recent significant loss with those who had not. Overall, three main findings emerged. First, resilience levels did not differ significantly between participants who reported a recent loss and those who did not. Previous studies using the CD-RISC-25 have shown that scores vary across populations, with higher values typically observed in community samples compared to clinical populations [9]. In this context, the scores observed in the present study are consistent with a moderate level of perceived resilience. Compared with reference values reported in the original CD-RISC-25 study, the mean resilience score in our sample appears to be lower than that observed in general population samples and closer to levels reported in primary care or mixed community-clinical populations [9]. This difference may be partly explained by sample characteristics, including the relatively young age of participants and the high proportion of individuals who had experienced a recent significant loss, which may have influenced perceived resilience levels at the time of assessment. Second, within the bereaved subsample, resilience was associated with educational level and religiosity. Third, grief-related functional impairment showed significant associations with several contextual factors, including sex, relationship to the deceased, time since loss, perceived social support, and psychotropic medication use.

One of the most notable findings of this study is that self-reported resilience, as measured by the CD-RISC-25, was similar among individuals with and without recent bereavement. However, this finding should be interpreted within the context of the demographic characteristics of the sample. Importantly, this finding reflects cross-sectional similarities observed at a single time point and should not be interpreted as evidence of stability over time. Additionally, the CD-RISC-25 primarily captures relatively stable, trait-like aspects of resilience, reflecting individuals’ perceived capacity to cope with adversity, rather than the dynamic and process-oriented nature of resilience over time. This result aligns with a substantial body of literature suggesting that many individuals demonstrate relatively stable psychological functioning after experiencing loss. Evidence suggests that resilience represents a common trajectory following potentially traumatic events, including bereavement, characterized by the capacity to maintain relatively stable psychological and social functioning despite adversity [8]. Longitudinal studies have similarly documented that many bereaved individuals adapt without developing significant psychopathology, highlighting the importance of differentiating between normative grief reactions and clinically significant impairment [8,19,20].

The absence of significant differences in resilience between bereaved and non-bereaved participants in the present study may reflect the adaptive coping mechanisms that many individuals mobilize following loss. Resilience is increasingly conceptualized not as a rare trait but as a dynamic capacity that enables individuals to maintain or regain psychological equilibrium in the face of stressors [21,22]. From this perspective, experiencing a loss does not necessarily imply diminished resilience, particularly when individuals possess psychological resources that facilitate adaptation.

At the same time, the results of this study suggest that resilience and grief-related impairment represent distinct psychological processes. Although participants with recent loss did not show lower resilience levels, the GIS revealed significant functional difficulties in a subset of individuals. This distinction supports theoretical models of grief that emphasize the multidimensional nature of bereavement responses [23,24,25,26,27,28,29,30,31,32,33,34,35]. For example, the dual process model of coping with bereavement proposes that individuals oscillate between loss-oriented and restoration-oriented processes as they adapt to the death of a loved one [26,27,28,29]. Under this framework, the presence of grief-related impairment does not necessarily imply a global reduction in resilience. Consistent with this interpretation, the absence of a significant correlation between resilience and grief-related functional impairment suggests that these constructs may operate relatively independently within the bereavement process. One possible explanation is that resilience, as assessed in this study, reflects individuals’ perceived capacity to cope with adversity, whereas grief-related functional impairment captures the extent to which grief interferes with daily functioning. As such, individuals may report adequate coping resources while still experiencing difficulties in specific functional domains. This apparent dissociation further highlights the complexity of bereavement responses and supports the need to assess both adaptive capacities and functional outcomes when evaluating adjustment to loss.

The present findings also highlight the importance of contextual and social factors in shaping grief-related outcomes. In the bereaved subsample, grief-related functional impairment was significantly associated with sex, type of relationship with the deceased, time since the loss, perceived social support, and psychotropic medication use. These associations are consistent with prior research indicating that the closeness of the relationship to the deceased is one of the strongest predictors of grief severity [30,31]. In particular, losses involving immediate family members, such as parents or siblings, are often associated with more intense grief reactions and greater functional impairment.

Similarly, the association between lower perceived social support and higher grief impairment observed in this study is well supported in the literature. Social support has long been identified as a protective factor in bereavement adjustment, buffering the psychological impact of loss and facilitating adaptive coping processes [32,33]. Individuals who perceive greater emotional and practical support from their social networks may be better able to process grief experiences and maintain daily functioning. However, this finding should be interpreted with caution, as perceived social support was assessed using a single-item measure that may not capture its multidimensional nature.

Another relevant finding concerns the association between religiosity and resilience among bereaved participants. Individuals who described themselves as fairly religious showed higher resilience scores compared with those who reported lower levels of religiosity. This result is consistent with research suggesting that religious and spiritual beliefs may provide meaning-making frameworks that help individuals interpret and cope with stressful life events, including bereavement [34,35]. Religious coping strategies may facilitate emotional regulation, foster hope, and offer existential explanations for loss, thereby promoting psychological adjustment [36,37,38]. This association should also be interpreted cautiously, as religiosity was assessed using a single-item measure that may not fully reflect its multidimensional nature.

Educational level also emerged as a significant factor associated with resilience in both the total sample and the bereaved subsample. Interestingly, the highest resilience scores were observed among participants with completed secondary education rather than those with higher educational attainment. Although this pattern should be interpreted cautiously, it may reflect differences in life experiences, coping strategies, or socioeconomic factors that were not directly measured in the present study. Previous research has suggested that resilience is shaped not only by formal education but also by broader psychosocial resources, including social support, life experience, and cultural factors [39]. However, given the limited overall model fit, this association should not be interpreted as evidence of a strong or independent effect. Similarly, the variation in the direction of effects across models may reflect differences in sample composition between the full sample and the bereaved subsample, as well as the limited explanatory power of the models, rather than consistent or generalizable patterns.

Importantly, the multivariable models explained only a modest proportion of the variance in resilience scores. This finding is not unexpected given the complex and multidimensional nature of resilience. Psychological resilience is influenced by a wide range of individual, social, and environmental factors, many of which were not included in the present analysis [40,41]. In particular, relevant variables such as prior trauma exposure, personality traits, socioeconomic status, and lifetime mental health conditions were not assessed, which may have contributed to the limited explanatory power of the models and potential model misspecification. Additionally, the relatively wide confidence intervals observed in the regression analyses suggest limited precision of the estimates, which may be partly attributable to the sample size of the bereaved subsample.

Therefore, the relatively low explanatory power of the models likely reflects both the inherent complexity of the construct and the omission of relevant predictors, and the identified associations should therefore be interpreted with caution. In this context, the findings should be considered exploratory in nature and warrant replication in larger and more diverse samples. Taken together, these findings contribute to a growing body of research examining the interplay between resilience and functional outcomes in bereavement.

### 4.1 Strengths

This study has several strengths that contribute to the literature on bereavement and psychological resilience. First, it represents one of the few empirical studies examining the relationship between resilience and grief-related functional impairment in a Latin American context, specifically in Paraguay. Research on bereavement processes remains heavily concentrated in North America and Europe, and studies conducted in other cultural contexts are essential for understanding how sociocultural factors influence grief experiences.

Second, the study employed validated psychometric instruments to assess both resilience and grief-related functional impairment. The use of the CD-RISC-25 and the GIS allowed for a multidimensional evaluation of adaptive and maladaptive responses to loss. Both instruments demonstrated high internal consistency in this sample, supporting the reliability of the measurements.

Third, the study incorporated contextual variables such as religiosity, perceived social support, relationship to the deceased, and time since loss. Including these factors provides a broader understanding of the psychosocial conditions that may influence resilience and functional impairment following bereavement. This approach aligns with contemporary models that conceptualize grief as a complex biopsychosocial process rather than a purely emotional reaction.

### 4.2 Limitations

Several limitations should be considered when interpreting the findings of this study. First, the cross-sectional design does not allow for causal inferences regarding the relationship between resilience and grief-related functional impairment. In addition, it does not allow differentiation between pre-existing levels of resilience and post-loss adaptation, nor does it permit the examination of temporal changes in resilience following bereavement. Longitudinal studies would be necessary to examine how resilience trajectories evolve over time following bereavement and to determine whether resilience predicts long-term adaptation to loss. Furthermore, the sample size estimation was based on an expected prevalence of recent bereavement that differed from the proportion observed in the final sample, which should be taken into account when interpreting the representativeness of the findings.

Second, the sample consisted predominantly of young adults with university-level education. In addition, participants were recruited through a non-probability convenience sampling strategy using online platforms, which may have introduced a degree of selection bias. This demographic profile may limit the generalizability of the findings to other age groups, socioeconomic contexts, or cultural environments. Specifically, the results may not be fully generalizable to men, individuals with lower levels of educational attainment, or older adult populations. In particular, the results should be interpreted as primarily reflecting patterns observed among young, highly educated adults in Paraguay, and may not be directly applicable to older individuals, men, or those with lower levels of education.

Third, all measures were based on self-report instruments, which may be subject to response biases such as social desirability or recall bias. Although the scales used in this study demonstrated strong psychometric properties, future research could complement self-report data with clinical interviews or longitudinal assessments. In addition, psychotropic medication use was assessed as a dichotomous variable without detailed information on type, dosage, or duration, which may limit the interpretation of the observed associations. This simplified measurement approach may also have contributed to residual confounding and should be considered when interpreting the observed relationships involving medication use.

Additionally, perceived social support and religiosity were assessed using single-item measures, which may not adequately capture the multidimensional nature of these constructs and may introduce measurement error. These variables should therefore be interpreted as simplified indicators rather than comprehensive assessments of social support and religiosity.

Furthermore, the multivariable models explained only a modest proportion of the variance in resilience scores. This likely reflects the complex and multifactorial nature of resilience, which is influenced by numerous psychological, social, and environmental factors that were not fully captured in the present study, including potentially relevant variables such as prior trauma exposure, personality traits, socioeconomic conditions, and mental health history. This may have resulted in residual confounding and limits the ability to fully explain variability in resilience outcomes.

Finally, the absence of adjustment for multiple comparisons in the univariate analyses may have increased the risk of Type I error, and the findings should therefore be interpreted with caution.

### 4.3 Clinical and Research Implications

The findings of this study have several implications for both clinical practice and future research. First, the observation that resilience levels did not differ significantly between individuals with and without recent bereavement suggests that many individuals retain adaptive psychological resources even after experiencing a significant loss. This highlights the importance of not assuming psychological vulnerability solely on the basis of bereavement status. At the same time, structured assessment tools may help clinicians identify individuals who experience meaningful functional impairment despite maintaining adaptive coping capacities, particularly in cases where grief-related difficulties affect daily functioning [42]. In routine clinical practice, this assessment could be incorporated through brief screening questions or short standardized instruments administered during initial evaluations, allowing clinicians to capture both perceived coping capacity and functional impact early in the care process.

Second, the results indicate that resilience and grief-related functional impairment may represent partially independent dimensions of the bereavement experience. From a clinical perspective, this underscores the need to assess not only emotional distress but also the extent to which grief interferes with everyday functioning. Screening approaches that evaluate both adaptive resources and functional impairment may help clinicians better identify individuals who could benefit from psychosocial support or targeted interventions [43]. In this context, general supportive strategies, including psychoeducation, normalization of grief responses, and reinforcement of existing social support, may be appropriate for most individuals, whereas more structured or intensive interventions may be warranted for those presenting with elevated functional impairment.

Third, the associations observed between grief-related impairment and contextual factors such as social support, religiosity, and the nature of the relationship with the deceased suggest potential targets for psychosocial interventions. Strengthening social support networks and acknowledging the role of cultural and spiritual resources may contribute to more comprehensive approaches to bereavement care, particularly in community and primary care settings [44]. These findings may also inform stepped-care models of bereavement support, in which low-intensity, broadly accessible interventions are offered initially, with more specialized care reserved for individuals with persistent or severe functional impairment—an approach that may be particularly relevant in low-resource settings.

Finally, these findings highlight the importance of expanding research on resilience and grief-related functioning in underrepresented cultural contexts. Most empirical studies on bereavement have been conducted in North American and European populations. Increasing research in Latin American populations may help refine existing theoretical models of grief and resilience and improve the cultural relevance of bereavement support strategies [45].

## 5. Conclusions

The findings of this study suggest that self-reported resilience, as measured by the CD-RISC-25, did not differ between individuals with and without recent bereavement at the time of assessment, particularly among young, highly educated adults, even among individuals who report functional difficulties associated with grief. This distinction highlights the importance of evaluating both adaptive psychological resources and functional impairment when assessing bereavement outcomes.

At the same time, contextual factors such as educational level, religiosity, social support, and the nature of the relationship with the deceased appear to play an important role in shaping grief-related experiences. These results reinforce the view that bereavement is a multidimensional process influenced by individual and social factors.

Future research should explore longitudinal trajectories of resilience and grief-related functional impairment, as well as the cultural and contextual variables that shape bereavement responses in different populations. A better understanding of these processes may contribute to the development of culturally sensitive interventions aimed at supporting individuals experiencing significant loss.

## Data Availability

All data reported in this paper are available from the corresponding author upon request.

## Abbreviations

CD-RISC-25, Connor–Davidson Resilience Scale; GIS, Grief Impairment Scale; M, mean; SD, standard deviation; Min, minimum; Max, maximum; GVIF, Generalized Variance Inflation Factor.

## References

[1] Stroebe W, Stroebe M, Abakoumkin G, Schut H (1996). The role of loneliness and social support in adjustment to loss: a test of attachment versus stress theory. Journal of Personality and Social Psychology.

[2] Simon NM, Shear MK (2024). Prolonged Grief Disorder. New England Journal of Medicine.

[3] Zisook S, Shear K (2009). Grief and bereavement: what psychiatrists need to know. World Psychiatry : Official Journal of the World Psychiatric Association (WPA).

[4] Rubin SS, Malkinson R, Witztum E (2020). Traumatic Bereavements: Rebalancing the Relationship to the Deceased and the Death Story Using the Two-Track Model of Bereavement. Frontiers in Psychiatry.

[5] Lee SA, Neimeyer RA (2023). Grief Impairment Scale: A biopsychosocial measure of grief-related functional impairment. Death Studies.

[6] Caycho-Rodríguez T, Lee SA, Vilca LW, Lobos-Rivera ME, Flores-Monterrosa AN, Tejada Rodríguez JC (2023). A Psychometric Analysis of the Spanish Version of the Grief Impairment Scale: A Screening Tool of Biopsychosocial Grief-Related Functional Impairment in a Salvadoran Sample. OMEGA - Journal of Death and Dying.

[7] Caycho-Rodríguez T, Lee SA, Yupanqui-Lorenzo DE, Ventura-León J, Vilca LW, Baños-Chaparro J (2024). Cross-cultural validity of the Grief Impairment Scale (GIS): evidence from Four Latin American countries. Illness, Crisis & Loss.

[8] Bonanno GA (2004). Loss, trauma, and human resilience: have we underestimated the human capacity to thrive after extremely aversive events?. The American Psychologist.

[9] Connor KM, Davidson JRT (2003). Development of a new resilience scale: the Connor-Davidson Resilience Scale (CD-RISC). Depression and Anxiety.

[10] Broche-Pérez Y, Rodríguez-Martín BC, Pérez-Santaella S, Alonso-Díaz G, Hernández-Carballo A, Blanco-Consuegra Y, Broche-Pérez Y, Molerio-Pérez O (2012). Escala de Resiliencia de Connor-Davidson (CD-RISC). Validación de instrumentos psicológicos: Criterios básicos.

[11] Alim TN, Feder A, Graves RE, Wang Y, Weaver J, Westphal M (2008). Trauma, resilience, and recovery in a high-risk African-American population. The American Journal of Psychiatry.

[12] Wetherell JL (2012). Complicated grief therapy as a new treatment approach. Dialogues in Clinical Neuroscience.

[13] Boelen PA, Lenferink LIM (2020). Symptoms of prolonged grief, posttraumatic stress, and depression in recently bereaved people: symptom profiles, predictive value, and cognitive behavioural correlates. Social Psychiatry and Psychiatric Epidemiology.

[14] Torales J, Barrios I (2023). Diseño de investigaciones: algoritmo de clasificación y características esenciales. Medicina Clínica y Social.

[15] Gosling SD, Vazire S, Srivastava S, John OP (2004). Should we trust web-based studies? A comparative analysis of six preconceptions about internet questionnaires. The American Psychologist.

[16] Killgore WDS, Taylor EC, Cloonan SA, Dailey NS (2020). Psychological resilience during the COVID-19 lockdown. Psychiatry Research.

[17] Lwanga SK, Lemeshow S (1991). Sample size determination in health studies: a practical manual.

[18] Charan J, Biswas T (2013). How to calculate sample size for different study designs in medical research?. Indian Journal of Psychological Medicine.

[19] Bonanno GA, Kaltman S (2001). The varieties of grief experience. Clinical Psychology Review.

[20] Carr D, Boerner K, Moorman S (2020). Bereavement in the Time of Coronavirus: Unprecedented Challenges Demand Novel Interventions. Journal of Aging & Social Policy.

[21] Southwick SM, Charney DS (2012). The science of resilience: implications for the prevention and treatment of depression. Science (New York, N.Y.).

[22] Windle G (2011). What is resilience? A review and concept analysis. Reviews in Clinical Gerontology.

[23] Maciejewski PK, Zhang B, Block SD, Prigerson HG (2007). An empirical examination of the stage theory of grief. JAMA.

[24] Kaplow JB, Layne CM, Saltzman WR, Cozza SJ, Pynoos RS (2013). Using multidimensional grief theory to explore the effects of deployment, reintegration, and death on military youth and families. Clinical Child and Family Psychology Review.

[25] Hill RM, Oosterhoff B, Layne CM, Rooney E, Yudovich S, Pynoos RS (2019). Multidimensional grief therapy: Pilot open trial of a novel intervention for bereaved children and adolescents. Journal of Child and Family Studies.

[26] Stroebe M, Schut H (1999). The dual process model of coping with bereavement: rationale and description. Death Studies.

[27] Stroebe M, Schut H (2010). The dual process model of coping with bereavement: a decade on. OMEGA - Journal of Death and Dying.

[28] Fiore J (2021). A Systematic Review of the Dual Process Model of Coping With Bereavement (1999-2016). OMEGA - Journal of Death and Dying.

[29] Hall CA, Ungureanu I (2021). "Unbreak My Heart": Clinical Implications for Working With Bereaved Couples. The Family Journal.

[30] Prigerson HG, Horowitz MJ, Jacobs SC, Parkes CM, Aslan M, Goodkin K (2009). Prolonged grief disorder: Psychometric validation of criteria proposed for DSM-V and ICD-11. PLoS Medicine.

[31] Shear MK (2015). Clinical practice. Complicated grief. The New England Journal of Medicine.

[32] Burke LA, Neimeyer RA, McDevitt-Murphy ME (2010). African American homicide bereavement: aspects of social support that predict complicated grief, PTSD, and depression. OMEGA - Journal of Death and Dying.

[33] Aoun SM, Breen LJ, Howting DA, Rumbold B, McNamara B, Hegney D (2015). Who needs bereavement support? A population based survey of bereavement risk and support need. PloS One.

[34] Park CL (2005). Religion as a meaning‐making framework in coping with life stress. Journal of Social Issues.

[35] Dolcos F, Hohl K, Hu Y, Dolcos S (2021). Religiosity and Resilience: Cognitive Reappraisal and Coping Self-Efficacy Mediate the Link between Religious Coping and Well-Being. Journal of Religion and Health.

[36] Ano GG, Vasconcelles EB (2005). Religious coping and psychological adjustment to stress: a meta-analysis. Journal of Clinical Psychology.

[37] Pargament KI (2011). Spiritually integrated psychotherapy: understanding and addressing the sacred.

[38] Pargament KI, Exline JJ, Jones JW (2022). APA handbook of psychology, religion, and spirituality.

[39] Assarkhaniki Z, Rajabifard A, Sabri S (2020). The conceptualisation of resilience dimensions and comprehensive quantification of the associated indicators: A systematic approach. International Journal of Disaster Risk Reduction.

[40] Southwick SM, Bonanno GA, Masten AS, Panter-Brick C, Yehuda R (2014). Resilience definitions, theory, and challenges: interdisciplinary perspectives. European Journal of Psychotraumatology.

[41] Kalisch R, Cramer AOJ, Binder H, Fritz J, Leertouwer I, Lunansky G (2019). Deconstructing and Reconstructing Resilience: A Dynamic Network Approach. Perspectives on Psychological Science : a Journal of the Association for Psychological Science.

[42] Szuhany KL, Malgaroli M, Miron CD, Simon NM (2021). Prolonged Grief Disorder: Course, Diagnosis, Assessment, and Treatment. Focus (American Psychiatric Publishing).

[43] Killikelly C, Smith KV, Zhou N, Prigerson HG, O'Connor MF, Kokou-Kpolou CK (2025). Prolonged grief disorder. Lancet (London, England).

[44] Harrop E, Mann M, Semedo L, Chao D, Selman LE, Byrne A (2020). What elements of a systems' approach to bereavement are most effective in times of mass bereavement? A narrative systematic review with lessons for COVID-19. Palliative Medicine.

[45] Caycho-Rodríguez T, Valencia PD, Vilca LW, Lee SA, Carbajal-León C, Vivanco-Vidal A (2023). COVID-19 Bereavement in Ten Latin American Countries: Measurement Invariance of the Pandemic Grief Scale and Its Relation to Suicidal Ideation. OMEGA - Journal of Death and Dying.

